# Supplementary material for: Antibiotic resistant bacteria and commensal fungi are common and conserved in the mosquito microbiome
Source: PLoS One. 2019 Aug 14;14(8):e0218907. doi: 10.1371/journal.pone.0218907 (PMC6693846; doi:10.1371/journal.pone.0218907)
Supplement: S1 Table — The location of each site is indicated along with the two-letter code that is used to designate the site throughout the manuscript. (DOCX) [file pone.0218907.s001.docx]

| Nearest town | Trap location | Site Code | Lat./Long. (degrees, decimal min.) |
| --- | --- | --- | --- |
| Bethany | Bethany Bog | BB | 41° 26.708'N  72° 59.889'W |
| Middlefield | Durham Meadows | MI | 41° 29.508'N  72° 41.729'W |
| Shelton | Shelton Avenue | SH | 41° 18.179'N  73° 7.782'W |
| Southington | East Road/Kensington | SO | 41° 35.524'N  72° 50.513'W |
| Stafford | Nipmuck St. Forest | SF | 41° 58.196'N  72° 15.357'W |
| Wethersfield | Goff Road | WE | 41° 41.609'N  72° 42.025'W |

**Table S1.**  Location of the collection sites in the state of Connecticut.
