# Supplementary material for: Antibiotic resistant bacteria and commensal fungi are common and conserved in the mosquito microbiome
Source: PLoS One. 2019 Aug 14;14(8):e0218907. doi: 10.1371/journal.pone.0218907 (PMC6693846; doi:10.1371/journal.pone.0218907)
Supplement: Attachment — Submitted filename: Response to Reviewers.docx [file pone.0218907.s004.docx]

**Response to Reviewers**

We would like to thank the editor and reviewers for their comments. Please see our point-by-point response to the reviewers’ comments below.

**Academic editor**

Both reviewers are positive about your manuscript. I leave it p to you if you want to have the fungi included yes or no. Please reply to the other comments.

**Response:**

We have decided to leave the fungal data in the manuscript and our reasoning is described in the comments to reviewer 1 (see comment #1).

1. In your Methods section, please provide additional information regarding the permits you obtained for the work. Please ensure you have included the full name of the authority that approved the field site access and, if no permits were required, a brief statement explaining why.

**Response:**

We have added the following to the methods in order to document the authority under which the field samples were collected.

line 129 “Mosquitoes were collected as part of the state of Connecticut Mosquito Trapping and Arbovirus Surveillance Program. The Connecticut Mosquito Trapping and Arbovirus Surveillance Program was established by Public Act 97-289 in 1997”

**Reviewer #1:**

This reviewer enjoyed very much in reading this excellent manuscript. This work combines the fields of the vector-borne diseases, antimicrobial resistance and microbiology. The manuscript is well written, and the conclusion is well supported by the data.

**Response:**

We would like to thank the reviewer for their kind comments.

Main comments:

1) I strongly feel like that the portion of commensal fungi can be left out for another independent publication. Please focus on the beauty of this work, antimicrobial resistance and mosquitoes. Do not get distracted by the addition of fungi, in which you did not get any isolates, and cannot characterize the nature of their antimicrobial resistance;

**Response:**

We feel that the fungal data should remain in the current manuscript. Our reasoning is as follows:

These data are based on isolates. As we state in line 356 “through our efforts to culture antibiotic resistant bacteria we opened up a niche to culture fungal isolates from the mosquitoes”. We hypothesize that we recovered these fungal isolated by reducing the bacterial load on the antibiotic plates. We did not actually set out to culture fungi. In this regard our base media (LB) was not optimized for fungal recovery, as such the identification of fungi was a spurious finding in this study, not an objective. This data would not likely be sufficient for an independent manuscript.

2) For the field-caught mosquitoes, did you try to wash the mosquitoes before performing bacterial isolation etc? There are constant contact between mosquitoes and animal hosts/environmental surfaces etc. The identified microbiome could be on the surface of the mosquitoes, or their inside? Please provide more information, and make meaningful discussion;

**Response:**
This is something that we took care to control for but failed to mention in the methods. We have revised the methods to read “Prior to culturing individuals were washed in a solution of 90% ethanol to remove external adhering bacteria.” line 148

3) As indicated in Fig 2 (Schematic diagram of bacterial culturing), you used LB initially, followed by the utilization of different selective media. Please justify this approach. What would you get if you would use the selective media directly without the use of LB media? Even LB is not a type of selective media, the process of LB culture may still provide some preference of certain bacteria which overgrow over others;

**Response:**

The selective plates were LB plates supplemented with antibiotics. The purpose of this was to determine the proportion of antibiotic resistant cells in the “total” population. To avoid confusion we have added “LB media supplemented with antibiotics” to the figure legend of Figure 2 to make it clear that the media formulation is the same between all of the plates.

We agree with the reviewer that only using LB media likely influenced the bacteria that we recovered. We have added the following “However, as all culturing was performed on LB media these data likely only represent a proportion of the total bacterial diversity and favor fast growing bacterial species” to the results.

4) It is great that the authors used the bioinformatics to identify bacterial OUTs in this work. In the meantime, it would be more clinically more important and meaningful if you could identify the genus level and even species level of the isolated bacterial from the antibiotics-resistant plates. You are very close to provide this valuable information since these isolates are available to you, and you can even ID them by the use of the whole-length 16S rRNA sequencing.

**Response:**

While we agree with the reviewer that full-length sequencing could improve some of the ability to discern OTU’s, we argue that performing full length sequencing is not warranted for the following reasons.

1. Even full-length 16S rRNA gene sequencing is not generally considered to be sufficient to differentiate species or strains. This level of discrimination is generally performed by multi-locus sequencing, DNA-DNA reassociation kinetics, or whole genome sequencing, which is beyond the scope of this study.

2. Even with the short fragment that we sequenced ~75% of the isolates were classified to genus with >80% confidence (line 232). In this respect, there is likely to be little improvement in our data with full-length gene sequencing.

3. We have dedicated a section of our discussion (line 356) to discuss the potential limitations of using a short sequence fragment, so have already addressed this concern in the manuscript.

Reviewer #2: Well done paper.
On line 95 I think you are missing the word resistant - it states antibiotic populations and I think you meant to say antibiotic resistant populations. That is the only change that I have.

**Response:**

The suggested change has been made.
